# Zinc Fertilization Mitigates Boron Toxicity in *Citrus*: Plant Growth, Tissue Mineral Composition and Antioxidant Enzyme Activity

**DOI:** 10.3390/plants15172580

**Published:** 2026-08-24

**Authors:** Xiongjie Lin, Hanqing Hu, Yanfang Zhang, Shouxing Wen, Jiali Zou, Xiaowei Zhao, Ling-Yuan Zhang, Guocheng Fan, Jiahui Xu, Jing-Hao Huang

**Affiliations:** 1 Fruit Research Institute, Fujian Academy of Agricultural Sciences, Fuzhou 350013, China; linxiongjie@faas.cn (X.L.); hanqinghu@126.com (H.H.); zhangyanfangyes@sina.com (Y.Z.); shouxingwen2005@163.com (S.W.);; 2 Fujian Key Laboratory of Plant Nutrition and Fertilizer, Fujian Academy of Agricultural Sciences, Fuzhou 350013, China; 3 College of Horticulture, Fujian Agriculture and Forestry University, Fuzhou 350002, China; 4 College of Traditional Chinese Medicine, Fujian University of Traditional Chinese Medicine, Fuzhou 350122, China; 5 Institute of Plant Protection, Fujian Academy of Agricultural Sciences, Fuzhou 350013, China; guochengfan@126.com

**Keywords:** boron toxicity, zinc fertilization, Citrus, antioxidant enzyme, nutrient interaction

## Abstract

Boron (B) toxicity severely restricts Citrus growth and productivity in high-B soil and irrigation water environments. Although exogenous Zinc (Zn) has been reported to mitigate B toxicity in plants, the underlying physiological regulation mechanisms—particularly in Citrus—remain incompletely understood. In this study, seedlings of two Citrus species differing markedly in B tolerance—*Citrus sinensis* (sweet orange, B-tolerant) and *C. grandis* (sour pomelo, B-sensitive)—were subjected to three boric acid treatments (10, 200, and 400 μM) and five ZnSO_4_ treatments (0.002, 0.05, 0.1, 0.2, and 0.25 mM) in a fully factorial design over 15 weeks. Comprehensive physiological assessments—including growth parameters, photosynthetic performance, mineral-nutrient profiling (in leaves and roots), and antioxidant enzyme activity—were conducted to decipher the mechanistic basis of Zn-mediated B detoxification. Results showed that 400 μM BA alone triggered severe B toxicity symptoms in the older leaves of *C. grandis*, whereas 0.25 mM Zn independently led to chlorosis symptoms similar to Fe deficiency in the apical leaves of both *Citrus* species. Either B or Zn toxicity affected photosynthesis in matured leaves. Exogenous Zn at 0.05–0.20 mM alleviated B toxicity symptoms in B-sensitive *C. grandis*, yet consistent alleviation of Zn toxicity by excess B was not observed across all measured variables. Severe B toxicity caused a significant reduction in Fe, N, P and Ca contents and a significant increase in Cu and Mn contents in *C. grandis* roots, yet it only led to a significant reduction in Mn, N, P and Ca contents in *C. grandis* leaves. Unlike this, B toxic treatments in *C. sinensis* resulted in a significant reduction in merely Fe content and a significant increase in Cu, Mn, N and K contents in roots, and a significant reduction in Mn and Ca contents in the leaves. Under basal B supply, 0.05–0.20 mM Zn supplementation dose-dependently increased root Zn, Cu and Mn contents as well as leaf Zn and Cu contents in both *Citrus* species, but concurrently decreased root Fe content and leaf Fe and Ca contents—additionally, in *C. grandis*, leaf N, P, K and Mg contents were further reduced. Regarding antioxidant responses in *C. grandis*, B toxicity significantly suppressed leaf CAT activity while enhancing APX and GPX activities. Under B toxicity conditions, 0.2 mM Zn supplement significantly restored CAT activity and suppressed APX and GPX activities. These antioxidant modulations were highly dependent on species identity and treatment combination, and were markedly more pronounced in B-sensitive *C. grandis*. Collectively, this work clarifies the physiological interplay between Zn and B in Citrus, uncovers divergent adaptive strategies employed by contrasting citrus species under combined B–Zn stress, and provides a mechanistic foundation for optimizing exogenous Zn application to mitigate B toxicity in citrus production.

## 1. Introduction

Boron (B) is an essential trace element for the normal growth and development of plants, playing crucial roles in cell wall structure, cell division, sugar transport, and devising metabolism processes [1]. However, due to its narrow optimal concentration range, both B deficiency and B excess are commonly observed in agricultural systems [2]. Either B deficiency or B excess can negatively impact plant growth and crop yield. Substantial inter-specific variation exists among crop species with respect to sensitivity to excess boron [3]. Excessive B uptake and accumulation, especially in the leaf tissues, can hinder plant development when concentrations exceed metabolic requirements. Although numerous studies have investigated boron deficiency and optimized field-level boron fertilization strategies in calcareous soils [4], comparatively limited attention has been directed toward zinc-mediated mitigation of B toxicity in Citrus species. The inhibition occurs through multiple mechanisms, including disruption to cell division and expansion, reduced photosynthetic efficiency, compromised cell membrane integrity, and induction of oxidative stress [1,5,6]. Toxic symptoms such as chlorosis and necrosis appear in crops like corn, wheat, and rice when soil B content exceeds 5 mg/kg [7].

Strategies for alleviating B toxicity in plants are usually divided into three categories: reducing the concentration of tissue B; decreasing cellular B activity; and enhancing plant physiological tolerance [8,9]. Soil B excess can be managed through leaching B with water, chelating B with triisopropanolamine (TIPA), or application of amendments [10]. When developing amendments to improve soil conditions or mitigate B toxicity to plants, interactions between B and elements such as calcium (Ca), sulfur (S), zinc (Zn), silicon (Si), and aluminum (Al) are frequently exploited [11]. Research indicates that supplying plants with elements capable of interacting with excessive B enhances antioxidant enzyme activities, thus alleviating B toxicity [8]. As a well-documented exogenous protectant, glycine betaine (GB) alleviates B-induced toxicity in plants through coordinated modulation of antioxidant metabolism and osmotic homeostasis [12]. The interaction between B and Zn can influence the absorption of other nutrients, thereby altering mineral composition and affecting various metabolic processes in plants. Appropriate application of B and Zn fertilizers significantly improves crop yield and quality in potatoes, tomatoes, rapeseed, and wheat [13,14,15,16].

*Citrus* belongs to evergreen subtropical fruit trees. B toxicity has been documented in several Citrus-growing regions, particularly where soils or water exhibit elevated B concentrations [17,18]. For example, a field survey conducted in Pinghe County, Fujian Province, China,—home to over 54,000 ha of ‘Guanximiyou’ Pomelo orchards—revealed that 74.84% of leaf samples exceed established critical thresholds for B accumulation [19]. In Citrus production systems, excessive B can impair the absorption and transport of nutrients in roots, reduce leaf photosynthetic activity, and increase the risk of juice vesicle granulation in fruits [19,20,21].

Zn has been shown in Sour orange (*Citrus aurantium*) to reduce excessive B uptake in soils and limit B transport to the aboveground parts [22]. In Newhall navel orange (*C. sinensis*) grafted onto trifoliate orange (*C. trifoliata*) rootstock, supplementation with Zn can reduce leaf B accumulation by 30–40% compared to Zn deficiency [23]. However, existing studies predominantly focus on the protective roles of organic osmolytes—particularly glycine betaine—in modulating wheat responses to B, whereas the mechanistic basis by which essential mineral elements such as Zn mitigate B-induced toxicity remains largely unexplored. Despite these observational advances, the fundamental physiological and biochemical mechanisms by which Zn mitigates B toxicity remain poorly understood in *Citrus*. Grounded in these interdisciplinary insights, the present study systematically evaluates the ameliorative efficacy of Zn supplementation against B toxicity in Citrus, elucidates associated alterations in B distribution, tissue mineral element profiles, and antioxidant metabolism, and identifies an effective Zn concentration range that mitigates B toxicity and reduces the risk of excessive Zn accumulation under controlled pot-culture conditions.

## 2. Results

### 2.1. Proper Level of Zn Effectively Alleviates B Toxicity Symptoms in Citrus Leaves

**Figure 1:**
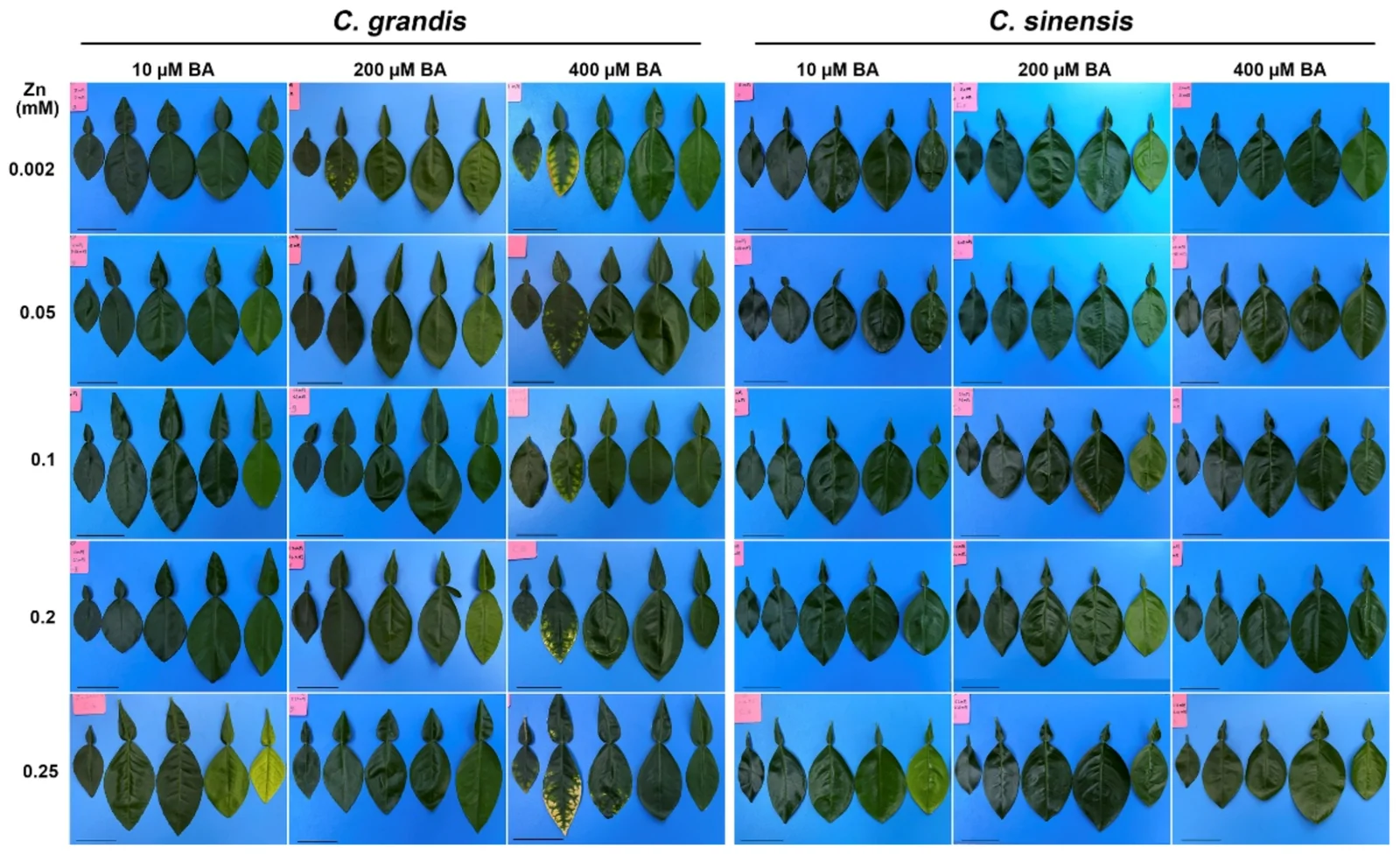
Phenotypic effects of B × Zn interaction on *C. grandis* and *C. sinensis* leaves. Leaves in each panel were collected from the basal, quarter-height, middle, three-quarter-height and top of the plants, which were treated with different levels of boron acid (BA) and zinc sulfate (Zn) for 15 weeks. Bars = 5 cm.

*C. sinensis* and *C. grandis* represent two *Citrus* plants showing different responses to B excess. Typical B toxicity symptoms, such as marginal and interveinal chlorosis, or brown and necrotic margins, were observed only in the leaves from lower plant parts (quarter-height to middle) of *C. grandis* seedlings under both moderate (200 μM) and severe (400 μM) B toxicity. Notably, these B toxicity symptoms disappeared when 0.05, 0.1, 0.2, or 0.25 mM zinc sulfate was applied under moderate B toxicity. Under severe B-toxic conditions, visible B toxicity symptoms were mitigated by supplementation with 0.05, 0.1 or 0.2 mM Zn sulfate; nevertheless, 0.25 mM Zn exacerbated interveinal chlorosis, even though necrotic symptoms were absent in leaf margins. Under basal B supply, 0.25 mM Zn treatment resulted in chlorotic apical leaves with a fine network of green veins in both Citrus species, which were characterized as typical Fe deficiency symptoms [24]. Notably, Zn-induced Fe deficiency chlorosis was more pronounced in *C. grandis* than in *C. sinensis*, indicating genotypic variation in metal nutrient homeostasis and root-to-shoot iron partitioning. Intriguingly, concurrent application of 200 or 400 μM B completely alleviated Zn-induced Fe deficiency chlorosis in both genotypes (Figure 1). This observation supports the plausible role of B–Zn interactions in modulating Fe bioavailability and utilization—through this remains a testable hypothesis requiring rigorous experimental validation.

### 2.2. Nutrient Element Contents in Leaf and Root Tissues

Under each given Zn concentration, increasing B supply significantly elevated leaf B content in both *Citrus* species. Notably, *C. sinensis* displayed higher leaf B concentrations compared with *C. grandis*. In contrast, leaf Zn content remained relatively stable across different B treatments. A three-way ANOVA was conducted to access the main and interactive effects of species, B treatment, and Zn treatment on leaf mineral compositions (Supplementary Table S1). A statistically significant three-way interaction among species, BA treatment and Zn treatment was observed for leaf Mn, Cu, N, P and Mg contents; in contrast, no significant three-way interaction was observed for leaf B, Zn, Fe, Ca or K contents. However, significant two-way interactions between BA treatment and Zn treatment were observed for leaf B, Cu, N, and Ca contents in both Citrus species; for leaf Fe, such an interaction was significant only in *C. sinensis*, and for P, K, and Mg contents, it was significant only in *C. grandis*. Significant two-way interactions between species and Zn treatment were detected for leaf Zn, Mn and Mg contents; in contrast, a significant interaction between species and BA treatment was observed only for leaf Cu and Mn contents.

**Figure 2:**
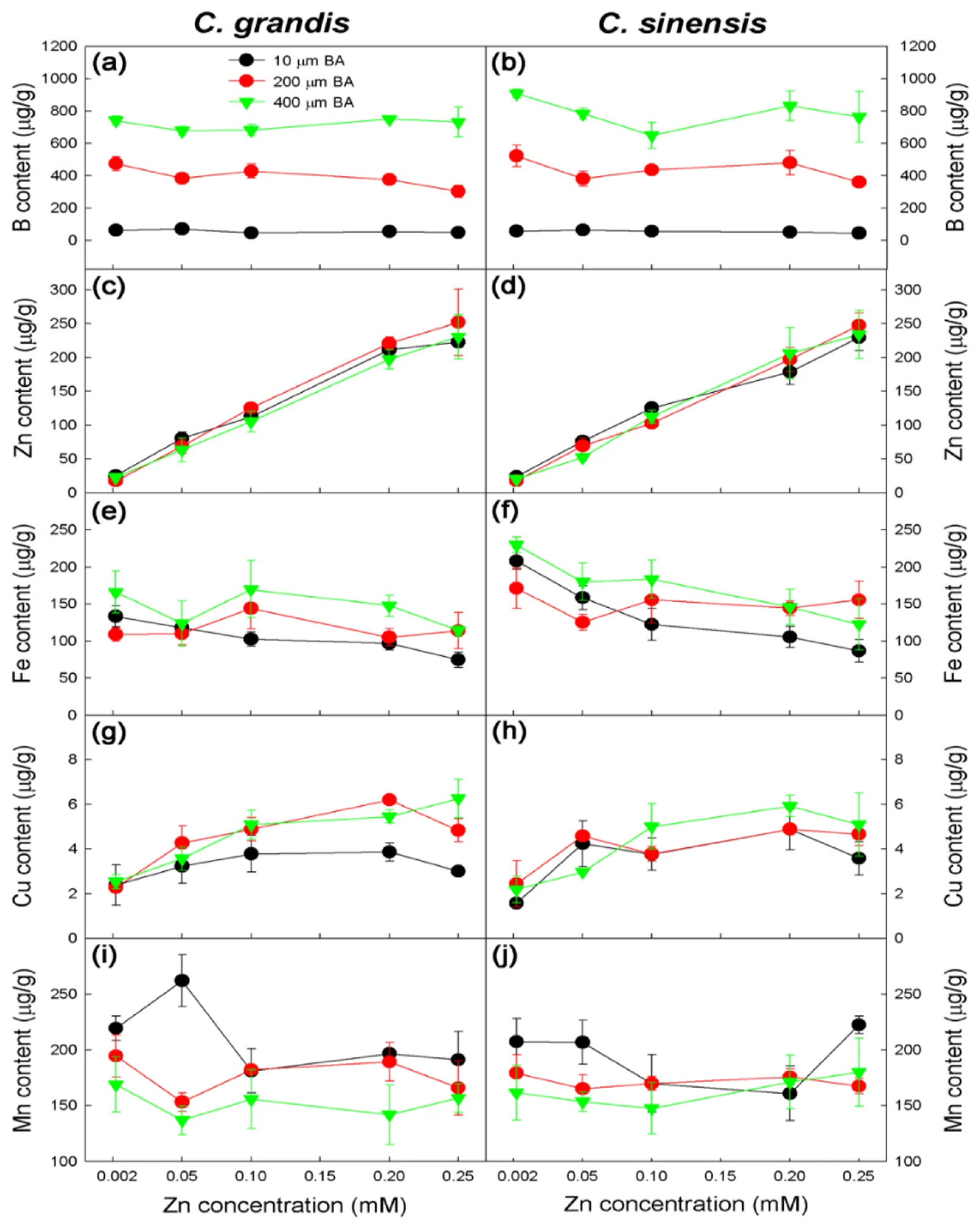
Effects of B × Zn interaction on micronutrient contents in leaves of *C. grandis* (**a**,**c**,**e**,**g**,**i**) and *C. sinensis* (**b**,**d**,**f**,**h**,**j**). Black circles, red circles and green inverted triangles represent 10, 200 and 400 μM BA treatments, respectively. Data are reported as mean ± SD of three independent pot-based biological replicates (*n* = 3).

Simple main effect analysis showed that 0.25 mM Zn treatment significantly reduced leaf B content under either moderate or severe BA stress in *C. sinensis* when compared with 0.002 mM Zn treatment, but only under moderate BA stress in *C. grandis* (Figure 2a,b). Nevertheless, Zn treatment had a simple main effect on leaf Zn content. Leaf Zn content in both *Citrus* species increased significantly with external Zn levels under each given BA treatment. No other main effects or interactions were obtained (Figure 2c,d). Further analysis showed that leaf Fe content decreased as the external Zn level increased under sufficient and severe toxic BA conditions in *C. sinensis*. Compared with sufficient BA treatment, moderate toxic BA significantly lowered Fe content in *C. sinensis* leaves under 0.002 mM Zn conditions, but increased it at 0.25 mM levels. Severe BA significantly increased Fe content in the leaves of both species (Figure 2e,f). With the exception of *C. grandis* under basal B supply, externally supplied Zn significantly increased leaf Cu content in both *Citrus* species, with values peaking at 0.2 mM Zn. Independent toxic BA treatment did not affect leaf Cu content (Figure 2g,h). Under basal Zn conditions, severe toxic BA treatment significantly decreased Mn content in the leaves of both *C. grandis* and *C. sinensis* (Figure 2i,j).

**Figure 3:**
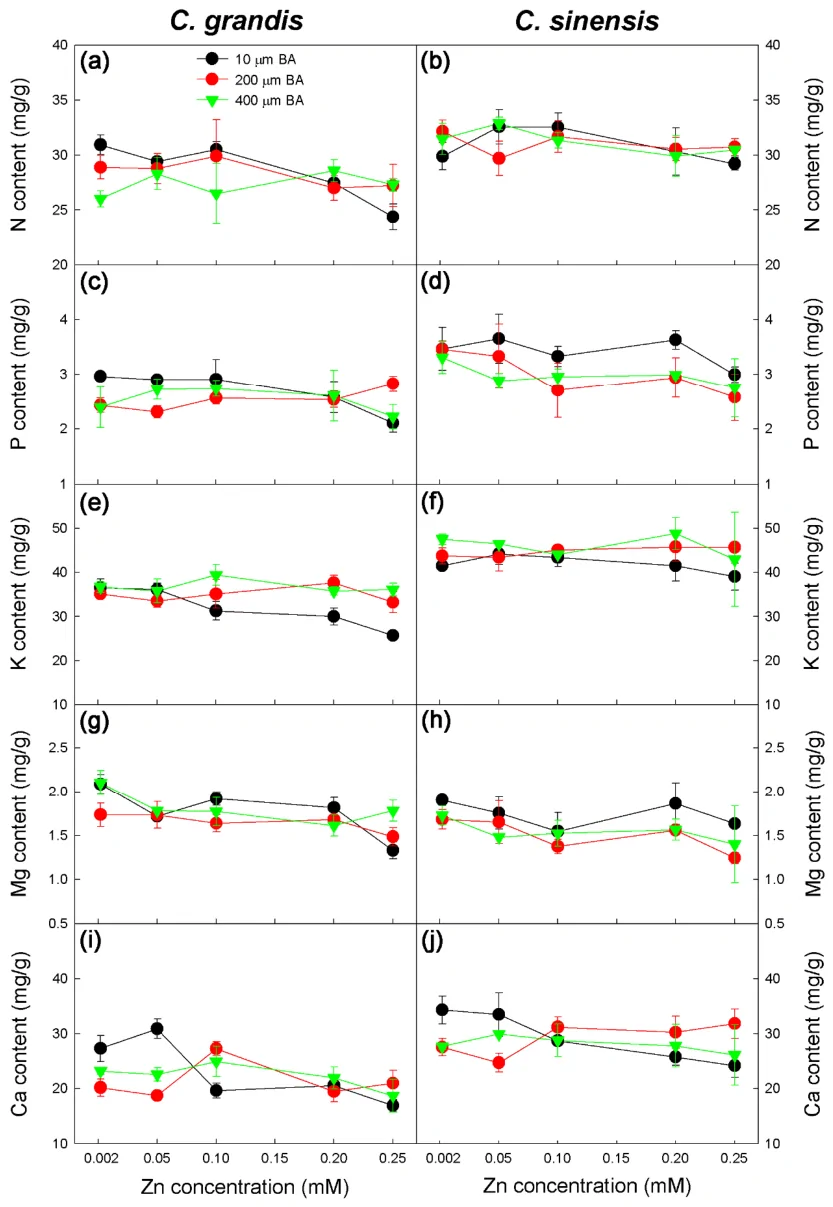
Effects of B × Zn interaction on macronutrient contents in leaves of *C. grandis* (**a**,**c**,**e**,**g**,**i**) and *C. sinensis* (**b**,**d**,**f**,**h**,**j**). Black circles, red circles and green inverted triangles represent 10, 200 and 400 μM BA treatments, respectively. Data are reported as mean ± SD of three independent pot-based biological replicates (*n* = 3).

Under basal B supply, 0.2 and 0.25 mM Zn treatments resulted in a significantly lower leaf N content than the control Zn treatment in *C. grandis*, and 0.25 mM Zn treatments led to lower leaf N content than the 0.05 and 0.1 mM Zn treatments in *C. sinensis*. No other group difference was found for moderate and severe toxic BA treatments (Figure 3a,b). Under basal B supply, 0.25 mM Zn treatment resulted in a significantly lower P content than 0.002, 0.05 and 0.1 mM Zn-treated leaves in *C. grandis*, whereas under moderate BA stress, 0.25 mM Zn treatment had a significantly lower P content than the 0.05 and 0.1 mM Zn treatments in *C. sinensis* (Figure 3c,d). Furthermore, 0.25 mM Zn treatment significantly reduced leaf K only in *C. grandis* (Figure 3e,f). Combined with 10 µM BA, 0.25 mM Zn significantly reduced Mg content in the leaves of C *grandis*, rather than *C. sinensis* (Figure 3g,h). Toxic BA treatments significantly lowered leaf Ca in either *C. grandis* or *C. sinensis*; under basal BA condition, over 0.1 mM of Zn levels significantly reduced leaf Ca in both species, and this reduction could be restored in *C. sinensis* under moderate toxic BA treatment (Figure 3i,j).

**Figure 4:**
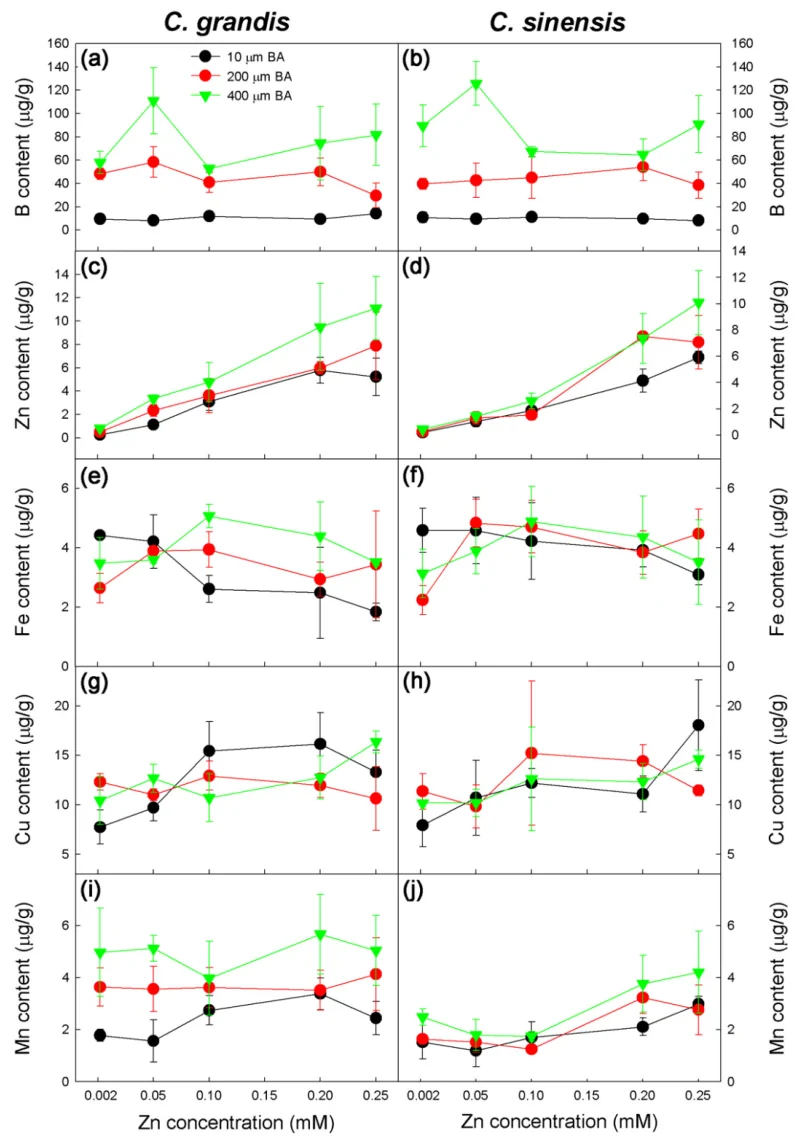
Effects of B × Zn interaction on micronutrient concentrations in roots of *C. grandis* (**a**,**c**,**e**,**g**,**i**) and *C. sinensis* (**b**,**d**,**f**,**h**,**j**). Black circles, red circles and green inverted triangles represent 10, 200 and 400 μM BA treatments, respectively. Data are reported as mean ± SD of three independent pot-based biological replicates (*n* = 3).

Similarly, root mineral composition was analyzed using three-way between-subjects ANOVA to assess the effects of species, BA treatment and Zn treatment, and their interactions, as well as inter-species differences (Supplementary Table S2). A statistically significant three-way interaction among species, BA treatment, and Zn treatment was detected only for root Cu concentration. However, significant two-way interactions between BA treatment and Zn treatment were observed for root B content in both *Citrus* species, whereas such interactions were detected only for root Fe, Cu, K, and Ca contents in *C. grandis*. Significant two-way interactions between species and Zn treatment were detected for root Mn, Mg, and Ca contents; in contrast, a significant interaction between species and BA treatment was observed for root B, Zn, Mn and Ca contents. BA treatment had a simple main effect on root B content in both *Citrus* species under each Zn treatment (Figure 4a,b). Unlike in the leaves, a significant two-way interaction between the effects of species and BA treatment for root Zn content was detected under 0.2 mM Zn treatment. Furthermore, 0.2 and 0.25 mM Zn treatments significantly increased root Zn content when compared with the basal Zn treatment under all BA conditions, and toxic BA treatment significantly increased root Zn content only when the external Zn level reached above 0.2 mM in both *Citrus* species (Figure 4c,d). A Zn level of 0.25 mM significantly decreased Fe content in *C. grandis* roots under basal B supply, rather than BA toxic stresses (Figure 4e,f). Under severe toxic BA stress, 0.25 mM Zn significantly increased Mn content only in *C. sinensis* roots (Figure 4i,j). Similar to the leaves, Zn treatment significantly increased root Cu content in both *Citrus* species in a dose-dependent manner. This Zn-dependent pattern was altered under moderate toxic BA stress, consistent with the significant B × Zn interaction (Figure 4g,h).

**Figure 5:**
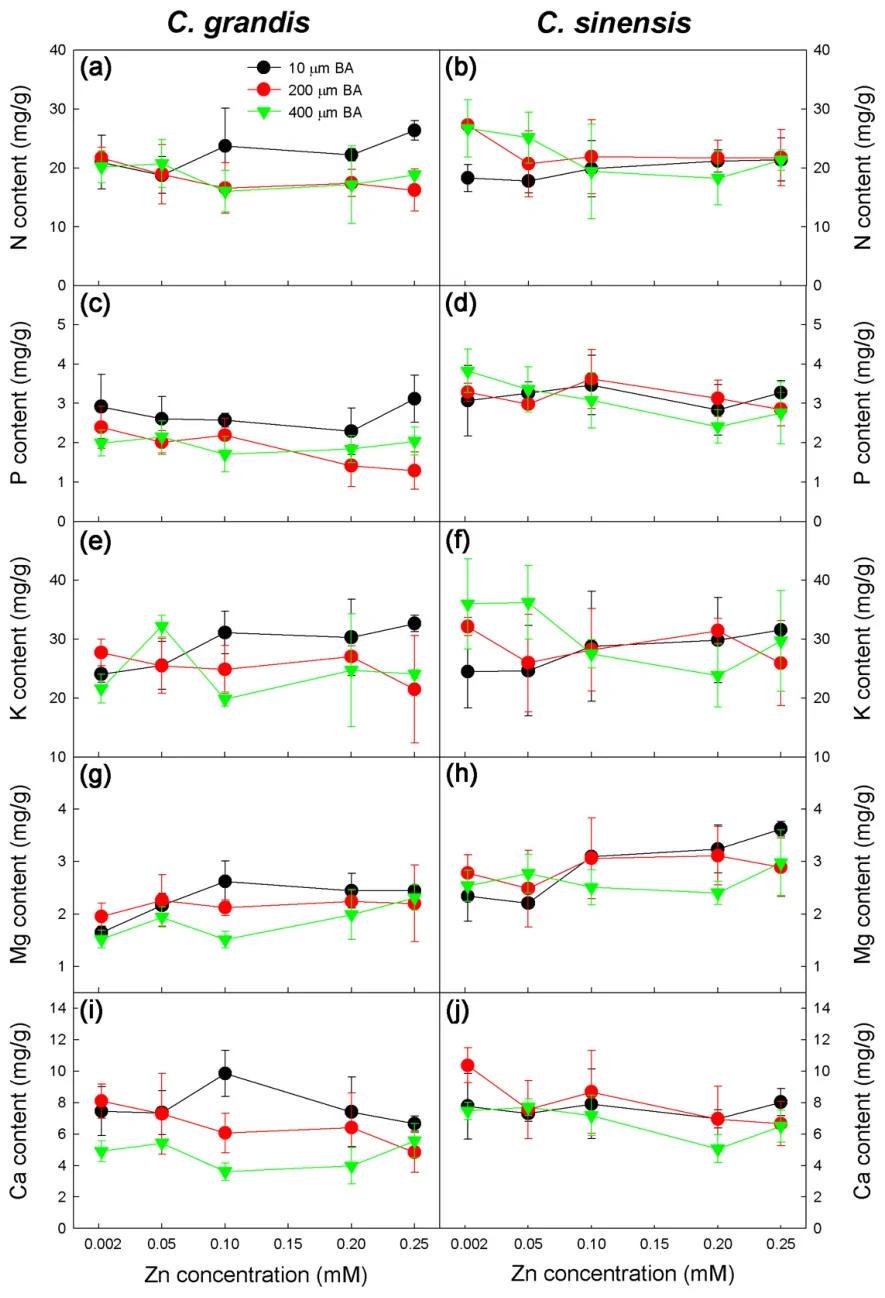
Effects of B × Zn interaction on macronutrient concentrations in the roots of *C. grandis* (**a**,**c**,**e**,**g**,**i**) and *C. sinensis* (**b**,**d**,**f**,**h**,**j**). Black circles, red circles and green inverted triangles represent 10, 200 and 400 μM BA treatments, respectively. Data are reported as mean ± SD of three independent pot-based biological replicates (*n* = 3).

Toxic BA treatments significantly decreased N and P content in *C. grandis* roots only under 0.25 mM Zn conditions, yet they increased N content in *C. sinensis* roots under basal Zn conditions (Figure 5a–d). Under basal B supply, 0.25 mM Zn resulted in a significantly higher root Mg content only in *C. sinensis*, compared to 0 and 0.05 mM Zn treatments (Figure 5g,h). Severe toxic BA treatment significantly increased root K content in *C. sinensis* under basal Zn conditions (Figure 5e,f). Under moderate BA stress, 0.25 mM Zn-treated *C. sinensis* roots had significantly higher Ca content than the control group. However, under 0.1 and 0.2 mM external Zn conditions, toxic BA treatment significantly decreased Ca content in the roots of *C. grandis* (Figure 5i,j).

Moreover, elemental analysis of apical leaves revealed that *C. grandis* and *C. sinensis* exhibited statistically comparable concentrations of B, Mn, Cu, Zn, Ca, Mg, K, P and N under control conditions, except for Fe, which was significantly higher in *C. sinensis* (*p* < 0.05). Under 0.25 mM Zn treatment, leaf Zn concentrations increased markedly in both species: by 3.5-fold in *C. grandis* and by 4.2-fold in *C. sinensis*. Critically, at this Zn supply level, *C. sinensis* accumulated significantly more Zn than *C. grandis* (*p* < 0.05), indicating greater Zn uptake capacity. Zn application also induced significant declines in Fe, Ca, Mg, K and N concentrations in apical leaves of both species (all *p* < 0.05). In contrast, reductions in B and Mn were observed exclusively in *C. sinensis*, whereas P concentration decreased solely in *C. grandis*, and Cu concentration, conversely, increased significantly only in *C. sinensis* (Table 1; Supplementary Table S3).

### 2.3. Effects of B × Zn Interaction on Photosynthesis

**Figure 6:**
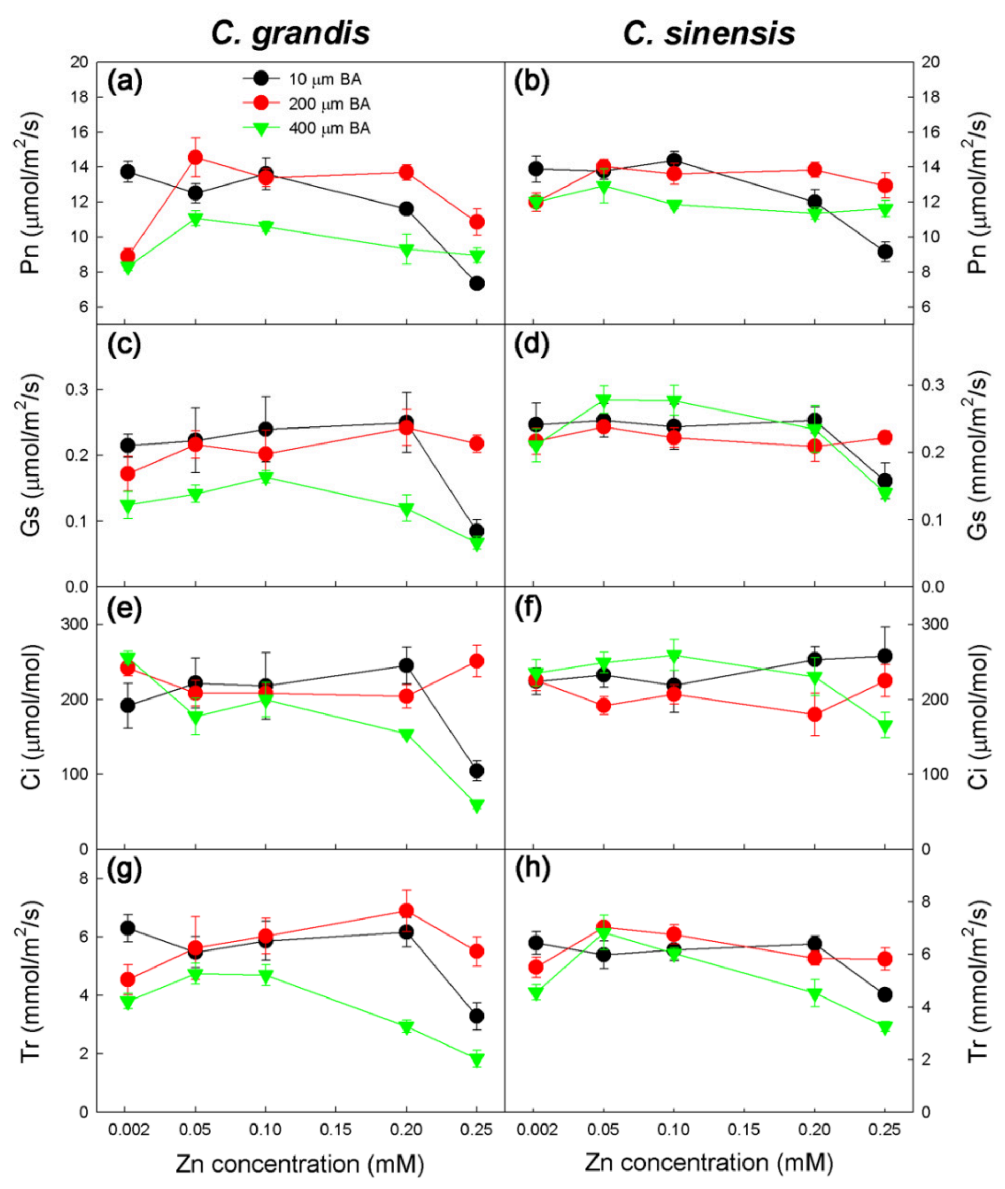
Effects of B × Zn interaction on photosynthetic parameters in *C. grandis* (**a**,**c**,**e**,**g**) and *C. sinensis* (**b**,**d**,**f**,**h**). Black circles, red circles and green inverted triangles represent 10, 200 and 400 μM BA treatments, respectively. Data are reported as mean ± SD of six independent pot-based biological replicates (*n* = 6). Pn, net CO_2_ assimilation rate; Gs, stomatal conductance; Ci, intercellular CO_2_ concentration; and Tr, transpiration rate.

The dependence of CO_2_ assimilation (Pn), stomatal conductance (Gs), intercellular CO_2_ concentration (Ci) and transpiration rate (Tr) differences between species on B leaf position was reflected in a significant species × BA treatment × Zn treatment interaction in an overall ANOVA of these data (Figure 6; Supplementary Table S4).

Moderate and severe toxic BA treatments significantly decreased Pn in both species, with *C. sinensis* having a significantly higher Pn than *C. grandis*. Under basal BA conditions, supplementation of Zn exceeded 0.2 mM and resulted in a significant decrease in Pn in both species, although this Pn decrease could be significantly improved by BA supplementation in the NS. Under moderate toxic BA stress, supplementation of Zn of no more than 0.2 mM could even restore the leaf Pn to control levels in both species (Figure 6a,b). Under basal B supply, Zn supply of ≤ 0.2 mM produced a non-significant rise in Gs in both species, where 0.25 mM Zn significantly reduced Gs (*p* < 0.05). Severe BA toxicity only significantly decreased Gs in *C. grandis*. Furthermore, 200 uM (rather than 400 uM) could restore Zn-toxicity-induced decreases in Gs in both *Citrus* species (Figure 6c,d). Single BA toxicity increased Ci merely in *C. grandis*. As external Zn level increased, leaf Ci slightly increased under basal B supply in both *C. grandis* and *C. sinensis*, although 0.25 mM Zn resulted in the rapid descent of Ci levels only in *C. grandis*. Under severe BA toxicity, 0.25 mM Zn intensified Ci reduction in both species (Figure 6e,f). BA and Zn toxicity alone significantly decreased Tr in both *Citrus* species. Zn-toxicity-induced Tr reduction could be recovered by moderate toxic BA treatment, but would be exacerbated by severe toxic BA treatment (Figure 6g,h).

### 2.4. Effects of B × Zn Interaction on Plant Biomass

**Figure 7:**
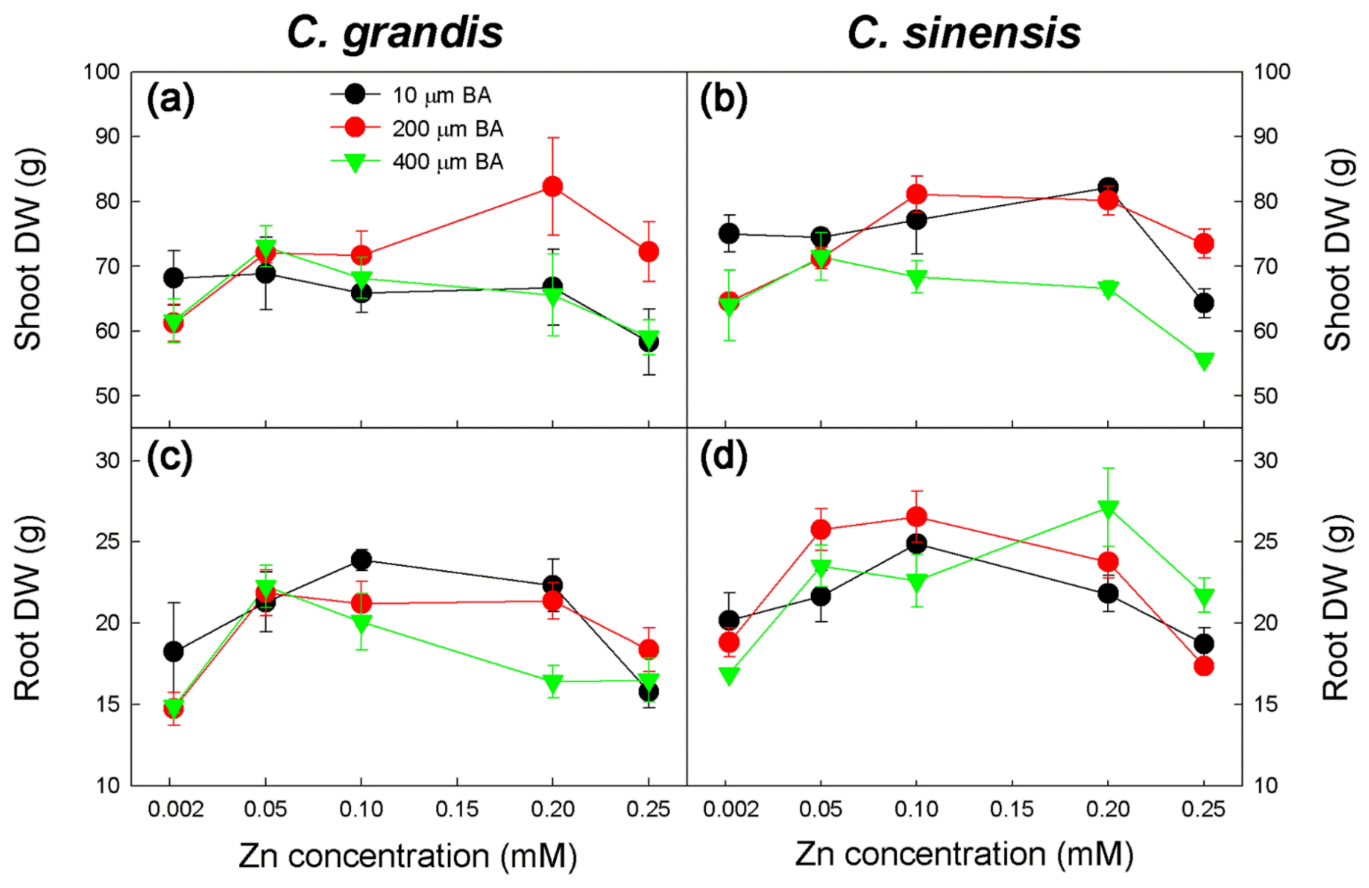
Effects of B × Zn interaction on shoot and root dry weight (DW) in *C. grandis* (**a**,**c**) and *C. sinensis* (**b**,**d**). Black circles, red circles and green inverted triangles represent 10, 200 and 400 μM BA treatments, respectively. Data are reported as mean ± SD of four independent pot-based biological replicates (*n* = 4).

No significant three-way interaction among species, BA treatment, and Zn treatment was detected for shoot dry weight (DW). However, significant two-way interactions were observed: (i) BA treatment × Zn treatment significantly affected shoot DW in both *C. grandis* and *C. sinensis*; (ii) species × Zn treatment exhibited significance under basal B supply and moderate B toxicity conditions; and (iii) species × B treatment was significant only under Zn supplementation of ≥0.1 mM. Notably, individual exposure to toxic BA treatments—or to toxic Zn treatment (0.25 mM) alone—significantly reduced shoot DW in both species. However, when BA and Zn co-occurred, this single-stress-induced reduction was ameliorated, with shoot DW recovering to near-control levels (Figure 7a,b; Supplementary Table S5).

A significant three-way interaction among species, BA treatment, and Zn treatment was detected for root DW. Significant two-way interactions were also observed: (i) BA treatment × Zn treatment significantly affected root DW in both *C. grandis* and *C. sinensis*; (ii) species × Zn treatment was significant under moderate and severe BA toxic conditions; and (iii) species × BA treatment was significant only under Zn supplementation ≥ 0.05 mM. Furthermore, Zn supplementation < 0.25 mM significantly increased root DW in *C. grandis* rather than *C. sinensis*. Moderate toxic BA treatment alone markedly reduced root DW in *C. grandis*, whereas severe toxic BA treatments induced a significant reduction in root DW in both *Citrus* species.

### 2.5. Effects of B × Zn Interaction on Antioxidant Enzyme Activities

**Figure 8:**
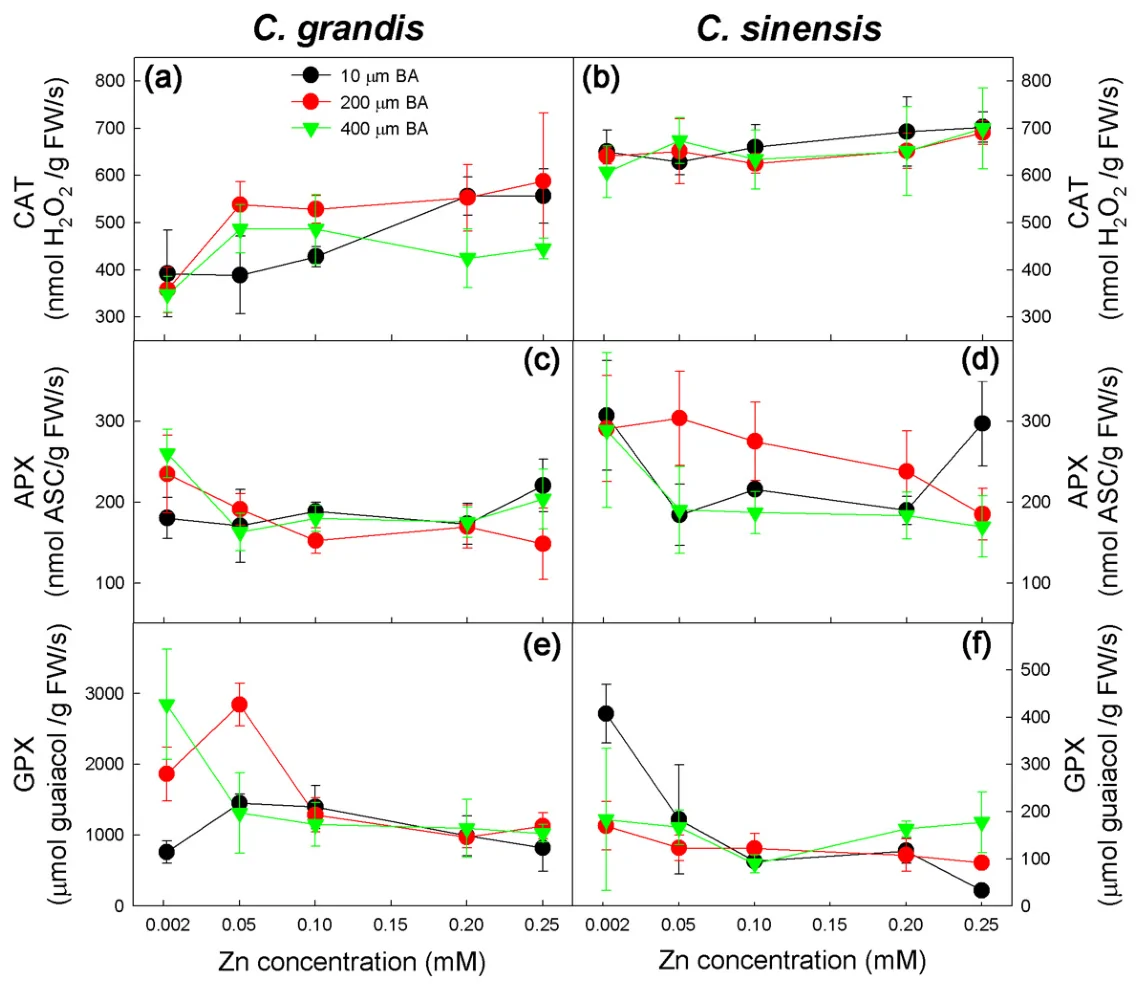
Effects of B × Zn interaction on APX, CAT and GPX activities in leaves of *C. grandis* (**a**,**c**,**e**) and *C. sinensis* (**b**,**d**,**f**). Black circles, red circles and green inverted triangles represent 10, 200 and 400 μM BA treatments, respectively. Data are reported as mean ± SD (*n* = 4).

Enzyme activity assays revealed that under 10 μM BA conditions, CAT and APX activities in *C. grandis* were significantly lower than those in *C. sinensis*, whereas GPX activity was significantly higher in *C. grandis* (Figure 8).

A significant three-way interaction among species, BA treatment, and Zn treatment was detected for CAT activity (Supplementary Table S6). Follow-up analysis identified significant two-way interactions: (i) BA treatment × Zn treatment in *C. grandis*; (ii) species × Zn treatment under basal B supply; and (iii) species × BA treatment under 0.25 mM Zn supplementation. A post hoc test indicated that 0.2 and 0.25 mM Zn significantly increased CAT activities under basal and moderate toxic BA conditions. Notably, CAT activity in *C. sinensis* leaves was significantly higher than that in *C. grandis* under each combined BA and Zn condition, except for the single 0.25 mM Zn toxic condition (Figure 8a,b).

No significant three-way interaction among species, BA treatment, and Zn treatment was observed for APX activity. However, significant two-way interactions were detected: (i) BA treatment × Zn treatment in *C. sinensis*; (ii) species × Zn treatment under basal B supply; and (iii) species × BA treatment under 0.05, 0.1 and 0.25 mM Zn supplementation. In *C. grandis* leaves, 0.25 mM Zn significantly decreased APX activity under moderate toxic BA stress, whereas only 0.05 mM Zn significantly decreased APX activity under severe toxic BA stress. In *C. sinensis* leaves, 0.05, 0.1 and 0.2 mM Zn significantly decreased APX activity under basal B supply, whereas 0.25 mM Zn significantly decreased APX activity under moderate toxic BA stress, and additional Zn supplementation significantly decreased APX activity under severe toxic BA stress. In contrast, independent toxic BA treatments significantly increased APX activity in *C. grandis* (Figure 8c,d).

A significant three-way interaction among species, BA treatment, and Zn treatment was observed for GPX activities. Significant two-way interactions included: (i) BA treatment × Zn treatment in *C. grandis*; (ii) species × Zn treatment under basal, moderate and severe BA toxic conditions (*F* = 17.689, *p* =0.001); and (iii) species × BA treatment under Zn supplementation ≤ 0.05 mM. Toxic BA treatments significantly increased GPX activity in *C. grandis* rather than *C. sinensis*. Moreover, Zn supplementation > 0.1 mM significantly lowered the BA-induced increase in GPX activity. *C. sinensis* had significantly lower GPX activity under all conditions except for the control (10 μM BA and 0.002 mM Zn; Figure 8e,f).

## 3. Discussion

In *Citrus* species, B exhibits limited phloem mobility, and B excess causes typical toxicity symptoms initially in older leaves, which progressively extend to younger leaves as stress intensifies [25]. Zn alleviates B toxicity primarily via two complementary mechanisms: enhancement of the antioxidant defense system and modulation of B transport [26]. This physiological behavior provides a foundation for investigating B × Zn interactions under B toxicity conditions. Short-term pot experiments demonstrated that exposure of Citrus seedlings to excessive B (200 µM) and Zn (0.25 mM) induces toxicity, whereas moderate supplemental Zn alleviates B toxicity by modulating mineral-nutrient uptake and translocation—particularly suppressing excessive B accumulation in leaves—and enhancing CAT activity, which is associated with improved leaf-level photosynthetic performance.

### 3.1. B × Zn Interaction in Plants

As an essential micronutrient, Zn contributes to root cell membrane stability and suppresses B absorption and transport [27]. The accumulation of B in plants increases under Zn deficiency [28], whereas it decreases when Zn fertilizers are applied [29]. Many scholars therefore believe that there is an antagonistic effect between B and Zn. However, a synergistic relationship between B and Zn has been reported in *Brassica campestris*, where excessive B exacerbates the symptoms associated with Zn toxicity [30]. Subsequently, López-Lefebre et al. [31] reported that B fertilization did not reduce the accumulation of Zn in *Nicotiana tabacum*. It seems that the interaction between B and Zn cannot be simply described as synergism or antagonism, since the B × Zn interaction in maize exhibits an antagonistic effect with respect to nutrient accumulation, yet exhibits a synergistic effect on growth [32]. These contradictions have been attributed to factors such as plant species, growth stage, and the severity of boron toxicity [26]. In B-sensitive *C. grandis*, Zn supply ranging from 0.05 to 0.20 mM effectively mitigated B toxicity symptoms (Figure 1), and promoted root growth under basal (10 µM) and BA toxicity (200 µM) conditions (Figure 7c), although 0.25 mM Zn exacerbated interveinal chlorosis caused by severe toxic BA treatment (Figure 1). It is noteworthy that, under sufficient and severe toxic BA treatments, the content of B in leaf and root tissues of *C. grandis* did not significantly change as Zn level increased (Figure 2a and Figure 4a); under toxic Zn conditions, the Zn content in leaves remained relatively stable across different BA treatments (Figure 2c), suggesting no direct interaction between B and Zn in terms of accumulation, and that mitigation of B toxicity by Zn does not depend on the reduction in tissue B concentration.

### 3.2. Crosstalk of Nutrient Elements

Crosstalk among mineral nutrients is well established at both morphological and physiological levels and is essential for maintaining plant mineral homeostasis [33,34,35]. Ionomic studies suggest that, under nutrient deprivation, the accumulation of other nutrient elements may be altered, thereby reshaping the mineral composition of plant tissues [36,37,38]. For instance, B deficiency suppresses phosphate uptake in *B. napus* [39]; N supply under high B conditions in canola promotes K and Ca accumulation [40]. Similar stress-induced imbalances in mineral homeostasis have also been reported in grapevines and wheat [41,42].

Despite the broad conservation of such nutrient interactions across plant species, mechanistic insights into B–Zn crosstalk remain scarce—particularly in Citrus. In this study, we identified genotype-specific B–Zn interactions in *C. grandis* and *C. sinensis*. Excess BA supply induced genotype-specific disruptions in mineral homeostasis, with contrasting responses observed between the roots and leaves of *C. grandis* and *C. sinensis*. In *C. grandis*, toxic BA caused widespread alterations in leaf macronutrient concentrations and significantly perturbed multiple root micronutrient levels—indicating pronounced antagonistic interactions among mineral elements. In contrast, mineral shifts under excess BA stress were markedly attenuated in *C. sinensis*. Notably, root elemental responses diverged sharply between genotypes: excess BA increased Cu and Mn accumulation in *C. grandis* roots, whereas *C. sinensis* roots exhibited elevated concentrations of Cu, Mn, N, and K. Collectively, these findings demonstrate that the extent and tissue specificity of B-induced mineral imbalance are genetically determined. Mn is well documented to antagonize iron (Fe) uptake in multiple plant species. As illustrated in Figure 4i, one plausible mechanistic hypothesis is that toxic boron acid (BA) impairs Fe acquisition and translocation by disrupting the Zn–Mn synergistic interaction—potentially contributing to Fe deficiency symptoms under high-B stress. However, this proposed pathway remains speculative; it has not been experimentally validated as a detoxification mechanism against Zn toxicity in Citrus, owing to the lack of direct functional evidence.

### 3.3. Zn Alleviation of B Toxicity in Citrus Is Concentration-Dependent

Zinc sulfate has been identified as the optimal zinc salt for mitigating B toxicity during adventitious root formation in mung bean, cucumber, and tomato [43]. The present study confirms that zinc sulfate effectively mitigates B toxicity in *C. grandis*, although with concentration dependency. Specifically, under moderate toxic BA conditions, Zn alleviated visible B toxicity symptoms (Figure 1), improved leaf photosynthesis (Figure 6a), and increased root and shoot biomass in a dose-dependent manner (Figure 7a,c). Surprisingly, co-application of BA and Zn at high concentrations (0.25 mM and 400 µM, respectively) produced opposite results compared to the mitigation effects observed at lower Zn concentrations, which was likely due to the excess Zn-induced imbalances of Cu (Figure 2g) and Ca (Figure 3i), resulting in significantly elevated leaf Cu concentrations and markedly reduced Ca concentrations relative to controls. Given that symptoms of Zn stress at high concentrations are not completely evident and might be dependent on species (Figure 1), our results provide a clear technical basis for optimizing Zn fertilization strategies. Under our greenhouse sand-culture experimental conditions, 0.1 mM Zn was effective in mitigating excess-B stress in Citrus seedlings. However, this concentration cannot be directly translated to commercial Citrus orchards. Field conditions—including soil chemistry, tree age, and rhizosphere properties—can substantially modulate Citrus responses to Zn supply. Consequently, controlled-environment findings must be validated under realistic orchard settings; additional field trials are therefore essential to translate these insights into robust, crop-specific Zn fertilization guidelines for commercial Citrus production.

### 3.4. Physiological Mechanisms Underlying Zn Mitigating B Toxicity in Citrus

It is well known that Zn serves as an essential cofactor for numerous enzymes and metalloenzymes critical to plant metabolism, including Cu/Zn-superoxide dismutase (SOD) and DNA-directed RNA polymerase, and participates in a variety of physiological processes [44]. Combined application of B and Zn fertilizers has been shown to mitigate oxidative stress induced by boron toxicity in many crops [15,26]. In plants, antioxidant enzymes such as CAT, APX and GPX play crucial roles in alleviating oxidative stress by scavenging reactive oxygen species (ROS). Nonetheless, the B-toxicity-induced modulation of APX, GPX, and CAT activity exhibited contrasting patterns between B-tolerant *C. sinensis* and B-sensitive *C. grandis* (Figure 8), precluding the definitive attribution of Zn-mediated B toxicity alleviation to any single antioxidant enzyme. Regarding Zn-dependent regulation of CAT activity: CAT does not utilize Zn as a catalytic cofactor. Critically, our study did not assess CAT gene expression, zinc-finger transcription factor binding affinity or occupancy at CAT promoters, CAT protein abundance, or post-translational modifications—thus, no direct molecular evidence for Zn-driven CAT regulation was generated. Drawing upon mechanistic insights from model and crop species (e.g., *Arabidopsis* and rice), two non-mutually exclusive, hypothetical pathways by which Zn may indirectly influence CAT expression and activity are proposed: (i) stabilization of cellular redox homeostasis, thereby preserving the structural integrity and functional competence of CAT and other antioxidant enzymes and (ii) structural support for zinc-finger transcription factors that recognize and bind cis-regulatory elements in CAT gene promoters to facilitate transcriptional activation. Nevertheless, neither pathway has been experimentally validated in Citrus under combined B–Zn stress; their relevance in this system remains speculative and requires targeted molecular investigation. However, this Zn-dependent transcriptional regulatory mechanism remains unconfirmed in Citrus under B–Zn interaction, given that our study did not perform CAT gene expression profiling or functional validation of zinc-finger transcription factor binding. In the present experiment, CAT activity was modulated under combined B–Zn stress; however, its utility as a reliable physiological marker for B-toxicity tolerance remains uncertain. Although direct evidence is lacking for Zn-dependent structural stabilization or functional modulation of CAT, Zn supplementation significantly increased CAT activity under toxic BA conditions (Figure 8a). Because neither H_2_O_2_ levels nor other oxidative-stress biomarkers were quantified in this study, the functional role of the observed CAT activity modulation in oxidative stress acclimation remains speculative. Regarding the decline in APX and GPX activities under Zn supply at 0.1 mM, we clarify that this reduction is not due to direct Zn-mediated enzymatic inhibition; 0.1–0.2 mM Zn supply significantly modulated APX and GPX activities under excess-B stress. One plausible hypothesis is that the observed decline in APX and GPX activities reflects a reduced cellular demand for ROS scavenging—consistent with attenuated oxidative pressure. However, this interpretation remains speculative: alternative mechanisms—including direct inhibition of APX/GPX enzymatic activity or post-translational regulation via altered protein abundance—cannot be ruled out. One plausible scenario is that the demand for APX- and GPX-mediated hydrogen peroxide scavenging decreases under alleviated B-toxicity, potentially triggering feedback-regulated, physiologically adaptive downregulation of these enzyme activities. If substantiated, such changes would reflect a functional recalibration of the antioxidant system—consistent with reduced oxidative pressure—rather than direct Zn-induced suppression. Collectively, this study provides a physiologically integrated perspective on how moderate Zn supply modulates Citrus responses to excess-B stress—revealing coordinated interactions across mineral homeostasis, leaf gas-exchange parameters (Pn, Gs, Tr), and antioxidant enzyme activities (CAT, APX, GPX).

Studies in non-Citrus plant species offer indirect molecular insights into B–Zn interactive responses. In strawberry, combined B–Zn treatments modulated the expression of genes associated with hormone signaling and sugar metabolism [45,46]. In *Arabidopsis thaliana*, the root-specific RING-family zinc-finger transcription factor gene RING1B (At1g03770) is transcriptionally induced under high-B stress and is postulated to regulate downstream B-responsive genes [47]. In barley, B toxicity triggered a two-fold upregulation of a C2H2-type zinc-finger transcription factor; similarly, RNA-seq analysis in wheat (*Triticum dicoccum*) revealed significant enrichment of three C3H-type zinc-finger protein genes (123068901, 123060371, and 123189473) in shoots under boron excess [48,49]. Collectively, these observations indicate that multiple zinc-finger transcription factor families—spanning RING, C2H2, and C3H subtypes—are evolutionarily conserved components of the plant transcriptional machinery deployed during boron stress adaptation. Nevertheless, none of these candidate genes have been functionally validated in Citrus, nor has their transcriptional responsiveness to Zn supplementation under B toxicity been examined. Crucially, it remains unknown whether exogenous Zn can drive transcriptional reprogramming of zinc-finger-related genes—and thereby contribute mechanistically to B detoxification—in Citrus seedlings.

## 4. Materials and Methods

### 4.1. Plant Materials

‘Sweet orange’ (*C. sinensis*; B-tolerant) and ‘Sour pomelo’ (*C. grandis*; B-sensitive) seedlings were cultivated following the protocol described by Han et al. [50]. Uniform six-week-old seedlings were transplanted into 6 L pots filled with fine river sand, with two plants per pot. The total experimental setup comprised 300 pots (10 biological replicates × 15 treatment combinations × 2 *Citrus* species) and pots were placed in a greenhouse under natural photoperiod and temperature conditions. Each pot received 500 mL of modified Hoagland’s nutrient solution (NS) every other day. The NS composition (pH 5.8, adjusted with dilute HCl or NaOH prior to application) was as follows: 6 mM KNO_3_, 4 mM Ca(NO_3_)_2_·4H_2_O, 2 mM NH_4_H_2_PO_4_, 1 mM MgSO_4_·7H_2_O, 10 µM H_3_BO_3_, 2 µM MnCl_2_·4H_2_O, 2 µM ZnSO_4_·7H_2_O, 0.5 µM CuSO_4_·5H_2_O, 0.065 µM (NH_4_)_6_Mo_7_O_24_·4H_2_O, and 20 µM Fe-EDTA. Eight weeks after transplantation, phenotypically uniform seedlings were equally randomly assigned to 15 treatment groups per species (three B levels × five Zn levels). There were 10 pots per treatment, where each pot represented one independent biological replicate. Each pot contained two seedlings, which served as subsamples within the same experimental unit, yielding 20 subsample plants in total per treatment. Each pot was irrigated with 500 mL of NS. All experimental pots were equipped with drainage holes at the bottom. To prevent salt accumulation in the sand substrate, each pot was leached weekly with deionized water throughout the 15-week treatment period. Treatments consisted of NS supplemented with boric acid (BA) to final levels at 10, 200, or 400 μM and ZnSO_4_·7H_2_O at 0.002, 0.05, 0.1, 0.2, or 0.25 mM. Each pot was irrigated with 500 mL of the corresponding treatment solution consecutively. The pH values of all the NS were adjusted to 5.8 with HCl or NaOH before applied. At harvest, fully expanded leaves were sampled from five vertical positions—basal, quarter-height, mid-canopy, three-quarter-height and apical—for comprehensive phenotypic assessment.

### 4.2. Photosynthetic Parameter Measurement

Photosynthetic parameters were measured on fully expanded, sun-exposed leaves located between one-quarter and one-third canopy height using a portable photosynthesis system (Li-6400XT, Li-COR Biosciences, Lincoln, NE, USA). Measurements were conducted between 9:00 a.m. and 11:00 a.m. under controlled environmental conditions: photosynthetic photon flux density (PPFD) of 1000 μM·m^−2^·s^−1^, ambient CO_2_ concentration of 400 μM·mol^−1^, and relative humidity of 60 ± 5%. Each measurement cycle was completed within 120 s to ensure physiological stability; six biological replicates (one plant per pot) were analyzed per treatment.

### 4.3. Determination of Mineral Composition

For mineral composition, three independent pot-based biological replicates (*n* = 3) were selected for each treatment. Within each selected pot, tissue samples from the two subsample seedlings were pooled to one composite sample for mineral quantification. Leaf samples (fully expanded, collected from one-third to mid-canopy height) and the whole root system were harvested. To assess potential Zn phytotoxicity, apical young leaves were additionally collected from the control (10 μM B + 0.002 mM Zn) and the highest Zn treatment (10 μM B + 0.25 mM Zn).

All tissues were thoroughly rinsed with deionized water, oven-dried at 75 °C for 72 h to constant weight, ground to a homogeneous fine powder, and sieved through a 60-mesh nylon screen. For elemental quantification, 0.15 g of sieved dried powder was accurately weighed into a PTFE microwave digestion vessel and digested with 5 mL of 65% (*v*/*v*) HNO_3_ and 1 mL of 30% H_2_O_2_. Samples underwent pre-digestion at room temperature for 30 min, followed by microwave-assisted digestion (MARS 6, CEM Corporation, Matthews, NC, USA) using a ramped temperature program: 120 °C (5 min) → 150 °C (5 min) → 180 °C (15 min). Digests were cooled to room temperature, diluted to 25 mL with ultrapure water (18.2 MΩ·cm), and filtered through 0.22 μm polyethersulfone membranes. Total N content was determined by high-temperature combustion using an elemental analyzer (Vario MAX CN, Elementar, Germany); concentrations of P, potassium (K), Ca, magnesium (Mg), Zn, B, iron (Fe), manganese (Mn), and Cu were quantified by inductively coupled plasma mass spectrometry (ICP-MS; Agilent 7900, Agilent Technologies, Santa Clara, CA, USA). Nutrient uptake (μmol·plant^−1^) was calculated as tissue nutrient content (μmol·g^−1^ DW) multiplied by dry weight (g).

### 4.4. Plant Biomass Measurement

At harvest, six representative pots per treatment were selected for biomass determination, with one seedling subsample from each pot. Shoots and roots were separated, placed in pre-labeled paper bags, and oven-dried at 80 °C for 72 h to constant weight. Following exclusion of pots affected by accidental seedling damage, four valid pot-based biological replicates (*n* = 4) remained and were used for subsequent biomass statistical analyses.

### 4.5. Extraction and Assay of Antioxidant Enzymes

For antioxidant enzyme assays, fresh leaf tissue (60 mg) was homogenized in 2.0 mL ice-cold 0.1 M phosphate buffer (pH 7.5) containing 1 mM ethylene-diamine-tetra-acetic acid (EDTA), 0.5% (*v*/*v*) Triton X-100, and 1% (*w*/*v*) polyvinylpolypyrrolidone (PVPP), following Chen et al. [24]. Homogenates were centrifuged at 12,000 rpm for 15 min at 4 °C; supernatants were collected and assayed immediately. Catalase (CAT; EC 1.11.1.6), ascorbate peroxidase (APX; EC 1.11.1.11), and guaiacol peroxidase (GPX; EC 1.11.1.7) activities were determined spectrophotometrically using established UV-absorption protocols [51], with absorbance changes monitored at 240 nm (CAT), 290 nm (APX) and 470 nm (GPX) using a UV spectrophotometer (TU-1810PC, Persee Analytical Instruments, Beijing, China). Four independent pot-based biological replicates (*n* = 4) were performed for each treatment.

### 4.6. Statistical Analysis

All data from the combined B–Zn toxicity experiments were analyzed using three-way analysis of variance (ANOVA), with Citrus species, B concentration, and Zn concentration as fixed main effects, along with all corresponding two-way and three-way interaction terms. For the apical-leaf dataset obtained under sole Zn treatment (i.e., in the absence of boron stress), a two-way ANOVA was conducted, treating Citrus species and Zn concentration as fixed factors and including their interaction term. All statistical analyses performed using IBM SPSS Statistics 22.0 (IBM Corp., Armonk, NY, USA). Graphical representations were generated using SigmaPlot 10.0 (Systat Software Inc., Palo Alto, CA, USA).

## 5. Conclusions

Results from the greenhouse sand-culture experiment demonstrate that 0.1 mM Zn supplementation effectively alleviates excess-B-induced physiological disturbances in Citrus seedlings—as evidenced by improved tissue mineral-nutrient profiles, leaf gas exchange parameters (Pn, Gs, Tr), and coordinated modulation of antioxidant enzyme activities (CAT, APX, and GPX). In contrast, the highest Zn treatment applied (0.25 mM) exacerbated adverse morphological and physiological symptoms under B stress. Critically, Zn-mediated alleviation of B toxicity was independent of reductions in tissue B concentration; although Zn supplementation altered the accumulation patterns of several mineral elements—including Ca, Mg, Fe, and Mn—no direct evidence supports the modulation of B uptake or translocation. Furthermore, Zn supplementation significantly enhanced CAT activity while suppressing APX and GPX activities, concomitant with improved leaf photosynthetic performance (Pn, Gs, Tr). Collectively, these findings indicate that, under the present greenhouse sand-culture experiment using root-zone Zn treatments, 0.1 mM Zn alleviate excess-B-induced physiological disorders in Citrus seedlings, whereas 0.25 mM Zn supplementation exerted phytotoxic effects on Citrus seedlings. One plausible hypothesis is that this toxicity stems from secondary iron deficiency—potentially driven by Zn-induced competitive inhibition of Fe uptake and root-to-shoot translocation. However, further research on the stability and sustainability of Zn fertilizers, as well as its long-term impacts on fruit quality and yield, are still needed to evaluate the practical effectiveness of B toxicity mitigation by Zn.

## Figures and Tables

**Table 1:** T Effect of Zn excess on mineral composition in apical leaves of two *Citrus* species.

Species	Treatment	B (μg/g)	Mn (μg/g)	Fe (μg/g)	Cu (μg/g)	Zn (μg/g)	Ca (mg/g)	Mg (mg/g)	K (mg/g)	P (mg/g)	N (mg/g)
*C. grandis*	Control	62.23 ± 11.51 ^a^	219.47 ± 11.08 ^a^	133.37 ± 14.13 ^b^	2.39 ± 0.90 ^bc^	25.10 ± 0.87 ^c^	27.82 ± 2.76 ^a^	2.09 ± 0.11 ^a^	39.72 ± 1.97 ^a^	3.36 ± 0.06 ^a^	30.93 ± 0.89 ^a^
	0.25 mM Zn	46.80 ± 1.05 ^a^	200.40 ± 31.70 ^a^	52.26 ± 1.52 ^c^	2.98 ± 0.51 ^b^	89.07 ± 3.53 ^b^	10.99 ± 1.28 ^b^	1.56 ± 1.00 ^b^	23.14 ± 0.21 ^b^	2.17 ± 0.10 ^b^	23.82 ± 0.95 ^c^
*C. sinensis*	Control	58.30 ± 6.46 ^a^	207.30 ± 20.96 ^a^	241.67 ± 46.28 ^a^	1.88 ± 0.46 ^c^	23.97 ± 1.19 ^c^	30.31 ± 3.51 ^a^	1.99 ± 0.02 ^a^	41.46 ± 0.73 ^a^	3.47 ± 0.39 ^a^	29.88 ± 1.21 ^a^
	0.25 mM Zn	32.63 ± 1.01 ^b^	152.10 ± 25.10 ^b^	60.23 ± 15.97 ^c^	4.57 ± 0.50 ^a^	99.53 ± 8.90 ^a^	14.36 ± 0.13 ^b^	1.63 ± 0.49 ^b^	20.40 ± 0.21 ^c^	3.68 ± 0.22 ^a^	26.84 ± 0.81 ^b^

Note: Different letters in the same column indicate a significant difference at *p* < 0.05 by two-way ANOVA (Bonferroni adjustment).

## Data Availability

The original contributions presented in this study are included in the article. Further inquiries can be directed to the corresponding authors.

## Supplementary Materials

The following supporting information can be downloaded at: https://www.mdpi.com/article/10.3390/plants15172580/s1, Table S1: Tests of Between-Subjects Effects for leaf mineral composition; Table S2: Tests of Between-Subjects Effects for root mineral composition; Table S3: Tests of Between-Subjects Effects for apical leaf mineral composition; Table S4: Tests of Between-Subjects Effects for photosynthesis; Table S5: Tests of Between-Subjects Effects for biomass; Table S6: Tests of Between-Subjects Effects for antioxidative emzyme activity.
